# Diagnosing and Treating Systemic Juvenile Idiopathic Arthritis and Adult-Onset Still’s Disease as Part of the Still’s Disease Continuum

**DOI:** 10.31138/mjr.290323.dat

**Published:** 2024-03-30

**Authors:** Apostolos Kontzias, Olga Petryna, Priscila Nakasato, Petros Efthimiou

**Affiliations:** 1 Department of Internal Medicine, Division of Rheumatology, Allergy and Immunology, Stony Brook University Hospital, Stony Brook, NY, USA,; 2 Department of Medicine, White Plains Hospital, White Plains, NY, USA,; 3 Novartis Pharmaceuticals Corporation, East Hanover, NJ, USA

**Keywords:** systemic juvenile idiopathic arthritis, adult-onset Still’s disease, biologics, autoinflammatory disease, macrophage activation syndrome

## Abstract

**Aim:**

We have summarised the existing evidence supporting the concept that systemic juvenile idiopathic arthritis (sJIA) and adult-onset Still’s disease (AOSD) are part of the same Still’s disease spectrum.

**Methods:**

A PubMed/Embase database search was conducted using specific search strings and free text words to screen for relevant articles. The search was limited to studies in humans, published up to June 2023, in English-language.

**Summary:**

sJIA and AOSD are rare autoinflammatory disorders that have similar pathophysiological and clinical features. The clinical presentations of sJIA and AOSD are highly variable, with differential diagnoses that include a broad range of malignancies, infectious diseases, and autoimmune disorders, which contribute to delays in diagnosis. Several sets of classification exist to help diagnose patients in clinical practice; the International League of Associations for Rheumatology criteria for sJIA and the Yamaguchi and Fautrel criteria for AOSD are the most-used criteria. The therapeutic strategy for Still’s disease aims to relieve signs and symptoms, prevent irreversible joint damage and potentially life-threatening complications, and avoid deleterious side effects of treatment. Recently, targeted therapies such as interleukin (IL)-1 and IL-6 inhibitors have become available for the treatment of sJIA and AOSD. While these biologics were originally largely reserved for patients in whom non-steroidal anti-inflammatory drugs, corticosteroids and conventional synthetic disease-modifying anti-rheumatic drugs had failed, they are increasingly used earlier in the treatment paradigm. Among IL-1 inhibitors, canakinumab is the only biologic approved in the US for the treatment of both sJIA and AOSD.

## INTRODUCTION

Systemic juvenile idiopathic arthritis (sJIA) and adult-onset Still’s disease (AOSD) are rare autoinflammatory disorders of unknown aetiology, characterised by a classic triad of arthralgia or arthritis, quotidian fever and a salmon-coloured rash.^1^ The annual incidence of sJIA is estimated to be 0.4–0.9 per 100,000,^2^ and of AOSD is 0.16 per 100,000.^3^ AOSD was first described in 1971 by Bywaters,^4^ who identified it as an inflammatory condition affecting young adults, typically 17–35 years old at disease onset. Bywaters observed similarities to sJIA, which was described earlier by George Still.^5^ Both are characterised by significant excess mortality, largely stemming from complications, particularly macrophage activation syndrome (MAS).^6^ A recent study of a United States nationwide inpatient database found that AOSD was responsible for 5820 hospitalizations in 2009–2013, with an inpatient mortality rate of 2.6% and the risk of in-hospital death significantly higher among Asian patients than White patients.^7^

The overlap in clinical features and pathophysiology between sJIA and AOSD suggests a shared disease continuum with different ages of onset.^1,8^ This review summarises the evidence in support of this continuum, along with diagnostic tools and targeted treatments.

## METHODS

A comprehensive literature search was performed in Medline/PubMed, Embase literature databases using the following MeSH terms, free text words, or search strings: “systemic juvenile idiopathic arthritis”, “adult-onset Still’s disease”, “sJIA”, “AOSD”, “sJIA and AOSD”, “AOSD and treatment”, “sJIA and treatment”, “canakinumab”, “AOSD and IL-1 inhibitors”, “sJIA and IL-1 inhibitors”, “AOSD and IL-6 inhibitors”, “sJIA and IL-6 inhibitors”, “Still’s disease”, “AOSD and TNF-α inhibitors”, “sJIA and TNF-α inhibitors”, and “Still’s disease and diagnosis”. The search was limited to studies in humans, published up to June 2023, in English-language. The reference lists of the articles identified in the PubMed/Embase search were also scrutinised for other potentially relevant articles.

## STILL’S DISEASE CONTINUUM

### Clinical manifestations

The concept of a Still’s disease continuum is based on the many clinical, genetic and laboratory features shared between sJIA and AOSD. Several studies have reported similar cardinal manifestations, laboratory features and a similar disease course in patients with sJIA and AOSD.^1,9^ Data suggest that sJIA and AOSD have a biphasic phenotype:^1,10–12^ an early phase, mainly characterised by systemic inflammation, and a later phase in which chronic polyarthritis prevails. In addition, patients with sJIA and AOSD are predisposed to MAS, a life-threatening complication^13–15^ that mimics conditions such as shock or multiorgan failure due to sepsis.^16^ It has been reported that 7–10% of patients with sJIA and 12–17% of those with AOSD develop MAS.^1^ Other potentially fatal complications reported in patients with Still’s disease include pulmonary arterial hypertension, myocarditis, disseminated intravascular coagulopathy, thrombotic thrombocytopenic purpura, amyloid A (AA) amyloidosis, acute respiratory failure, interstitial lung disease and alveolar proteinosis.^17–21^ Evidence suggests that various forms of lung disease can trigger systemic inflammation and, subsequently, MAS in patients with Still’s disease.^19,22,23^

### Pathogenesis

Interleukin (IL)-1 and IL-18 play a pivotal role in the pathogenesis of sJIA and AOSD.^1^ This is substantiated by the upregulation of IL-1α and IL-1β in healthy peripheral blood mononuclear cells^24^ and by improved AOSD outcomes after IL-1 inhibition.^25^ In addition, significant increases in serum levels of IL-18 and other cytokines have been observed in MAS.^26,27^ Similarly, it has been observed that although other cytokines normalise during remission, IL-18 remains elevated in inactive AOSD and sJIA.^28^

In addition to similarities in pathogenesis, sJIA and AOSD have similar genetic profiles. Gene-expression analyses have shown that genes that were downregulated after IL-1β inhibition in sJIA patients were conversely found to be upregulated in AOSD patients.^29,30^ These genes (e.g. IL-1β, IL-1 receptor accessory protein, IL-1 receptor antagonist protein, IL-1receptor, type I and IL-1 receptor, type II) that are upregulated in AOSD are all involved in IL-1 signalling pathways, indicating that AOSD is an IL-1–driven condition, mechanistically similar to sJIA. Polymorphisms of IL-6, IL-1α and IL1- receptor antagonist protein have been reported in patients with sJIA,^31–33^ and IL-18 polymorphisms have been reported in patients with AOSD.^34^ Macrophage migration inhibitory factor gene polymorphisms have also been observed in patients with sJIA^35^ and in those with AOSD.^36^

**Figure 1.**
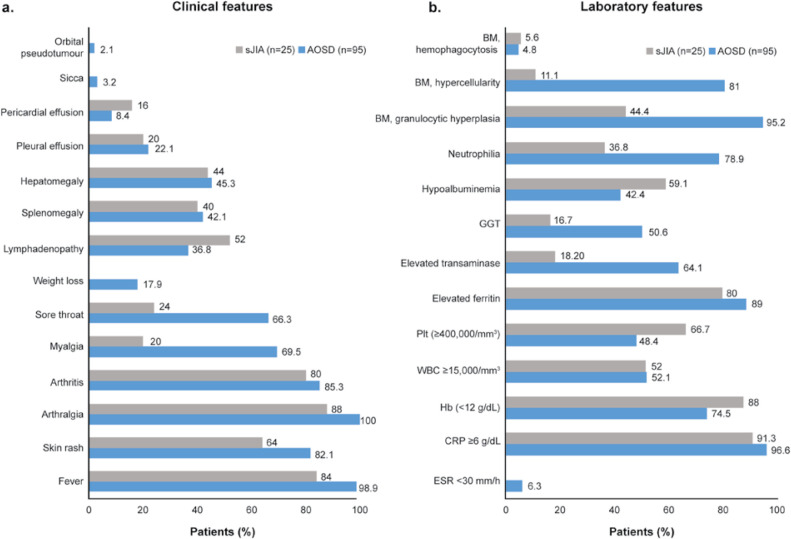
**(a)** Clinical and **(b)** laboratory features in patients with sJIA or AOSD. AOSD: adult-onset Still’s disease; BM: bone marrow microscopy; CRP: C-reactive protein; ESR: erythrocyte sedimentation rate; GGT: gamma-glutamyl transpeptidase; Hb: haemoglobin; Plt: platelets; sJIA: systemic juvenile idiopathic arthritis; WBC: white blood cells. [Source: Salih Pay, Nuran Türkçapar, Mukaddes Kalyoncu, Ismail Simşek, Esin Beyan, Ihsan Ertenli, M Akif Oztürk, Nurşen Düzgün, Hakan Erdem, Zeynep Ozbalkan, Sedat Kiraz, Gülay Kinikli, Nesrin Besbas, Ayhan Dinç, Aşkin Ateş, Umit Olmez, Meral Calgüneri, Olcay Tiryaki Aydintuğ, Ayşin Bakkaloğlu, Mustafa Turan, Murat Turgay, Yaşar Karaaslan, Rezzan Topaloğlu, Murat Duman, Seza Ozen; Ankara Rheumatology Study Group. Clin Rheumatol 2006;25(5):639–44]

Despite the many similarities, several differences between sJIA and AOSD have been identified. Unlike sJIA, AOSD is more common in females than males (70% vs 30%), suggesting a hormonal trigger for AOSD.^1,37,38^ While both conditions demonstrate seasonal variations, these are more pronounced in sJIA, possibly due to an immature immune system encountering antigens for the first time.^39,40^ In contrast, arthralgia/arthritis, sore throat, skin rash and myalgia are more common in AOSD,^9,41^ as are laboratory features including liver dysfunction, neutrophilia, granulocytic hyperplasia, and hypercellularity (**Figure 1**).

### Diagnosis

The diagnosis of Still’s disease is challenging due to the clinical heterogeneity of this rare condition.^42,43^ In the absence of a pathognomonic test, it is a clinical diagnosis of exclusion, with broad differential diagnoses of malignancy, infection and autoimmune disorders.^43,44^ The International League of Associations for Rheumatology (ILAR) criteria are widely used in the diagnosis of sJIA; these criteria require the presence of arthritis at presentation, documented quotidian fever of at least 2 weeks duration and any one of the following – serositis, organomegaly, evanescent rash and lymphadenopathy.^45^ For the diagnosis of AOSD, several classification criteria are used.^46–48^ These include Yamaguchi criteria,^47^ the most sensitive (96.2%) and widely used criteria, as well as Fautrel^46^ and Cush criteria48 (**Table 1**).^49^ Recently, ^50^ a revision of the ILAR criteria was proposed, which states that patients with sJIA might have systemic features with no arthritis, as seen in patients with AOSD.

**Table 1.** Commonly used diagnostic criteria for AOSD.

**Reference**	**Major criteria**	**Minor criteria**	**Diagnosis**
Yamaguchi M et al.^47^ (sensitivity, 96.2%; specificity, 92.1%)	Fever ≥39°C (≥1 week) Arthralgia (≥2 weeks) Typical rash Leucocytosis (≥10,000/mm^3^) including ≥80% of granulocytes	Sore throat Lymphadenopathy and/or splenomegaly Liver dysfunction Negative RF and ANA	Exclusions: Infections (especially sepsis and infectious mononucleosis) Malignancies (especially malignant lymphoma) Rheumatic diseases (especially polyarteritis nodosa and rheumatoid vasculitis with extra-articular features) Classification of AOSD requires: ≥5 criteria, including ≥2 major criteria
Cush JJ et al.^48^ (sensitivity, 84%)	Quotidian fever >39°C Still’s (evanescent) rash WBC count >12,000/mm^3^ + ESR >40 mm/h Negative RF and ANA Carpal ankyloses	Onset <35 years Arthritis Prodromal sore throat RES involvement or abnormal LFT results Serositis Cervical or tarsal ankylosis	Probable diagnosis: 10 points with 12 weeks of observation Definite AOSD: 10 points with 6 months of observation
Fautrel B et al.^46^ (sensitivity, 80.6%; specificity, 98.5%)	Spiking fever ≥39°C Arthralgia Transient erythema Pharyngitis PMN ≥80% Glycosylated ferritin ≥20%	Maculopapular rash Leucocytosis ≥10,000/mm^3^	Classification of AOSD requires: ≥4 major criteria; or 3 major criteria + 2 minor criteria

ANA: antinuclear antibodies; AOSD: adult-onset Still’s disease; ESR: erythrocyte sedimentation rate; LFT: liver function test; PMN: polymorphonuclear leucocytes; RES: reticuloendothelial system; RF: rheumatoid factor; WBC: white blood cell.

[Source: Kontzias A, Efthimiou P, Drugs, 2008;68(3):319–37.]

### Biomarkers

In light of the nature of Still’s disease as a diagnosis of exclusion, biomarkers can help rule out other conditions and avoid diagnostic delays.^51,52^ Higher serum ferritin levels (>1000 ng/mL) have been reported in patients with sJIA with MAS.[53] Serum ferritin level is often used as a biomarker to monitor disease activity and response to treatment in patients with AOSD.^54–56^ However, serum ferritin is considered less relevant for the monitoring of AOSD due to its limited specificity and the absence of a clear threshold level.^54^ The diagnostic value of glycosylated ferritin (GF) has also been studied. In healthy subjects, 50–80% of ferritin is glycosylated, but GF levels decrease to 20–50% in patients with inflammatory diseases.^57^ In AOSD, GF levels are low (≤20%), suggesting the involvement of additional mechanisms alongside inflammation.^54^ Fautrel et al.54 showed that a combination of hyperferritinaemia (>5 × upper limit of normal [ULN]) and a GF level of ≤20% yielded a specificity of 92.9% and a sensitivity of 43.2%.

Various cytokines have been investigated as biomarkers of Still’s disease, with several studies suggesting that IL-18 is a potential biomarker and an indicator of disease activity. A significant association between serum IL-18 levels and disease activity has been reported in patients with AOSD and in those with sJIA.^58^ In patients with sJIA, serum IL-18 levels reflected disease activity and predicted the disease course.^59^ Furthermore, higher levels of IL-6 correlated with some clinical features, including fever and C-reactive protein (CRP), in patients with sJIA,^60^ with high serum levels of IL-1β and IL-6 also being found in patients with AOSD.^61^

In addition, serum levels of soluble form of triggering receptor expressed on myeloid cells-1 (sTREM-1) were increased in AOSD suggesting that the initial elevation could predict a chronic course of AOSD.^62^ In patients with sJIA, serum concentrations of calcium-binding proteins S100A8/A9 correlated closely with disease activity and treatment response,^63^ whereas elevated levels of S100A12 were shown to distinguish sJIA from other causes of fever of unknown origin (FUO).^64^ Studies have also demonstrated a positive correlation between levels of S100A8/A9 and S100A12 proteins and disease activity and severity in patients with AOSD.^65–67^

Jia et al.^68^ observed that high levels of four circulating neutrophil extracellular traps (NETs; cell-free DNA, myeloperoxidase-DNA, neutrophil elastase-conjugated -DNA, and citrullinated histone 3 -DNA) correlated with levels of liver enzymes and inflammatory markers, as well as cardiopulmonary manifestations, suggesting the possible utility of circulating NETs as a biomarker of disease activity in AOSD. Recently, a study by Jung et al.^69^ identified that chemokine (C-C motif) ligand 2 levels in serum correlated with systemic score, leukocyte and neutrophil counts, and CRP, ferritin, lactate dehydrogenase, and albumin levels in patients with AOSD. A prospective observational study^70^ validated the neutrophil-to-lymphocyte ratio (NLR) of ≥4 as a biomarker of AOSD, with a sensitivity of 93.8%. The authors observed that the addition of NLR to the Yamaguchi and Fautrel classifications as a major criterion significantly improved their sensitivity while maintaining specificity. Other biomarkers, including procalcitonin and soluble CD163, have been evaluated in the assessment of disease activity in patients with Still’s disease, although their value in clinical practice has yet to be confirmed.^71–73^

#### Imaging

In patients with AOSD, narrowing of the intercarpal and carpometacarpal joint spaces have been observed, which can eventually lead to ankylosis. During the initial phase of the disease, radiographs are either normal or show slight joint space narrowing and, therefore, are usually not helpful for diagnostic purposes.^74,75^

#### Diagnostic Algorithm

**Figure 2.**
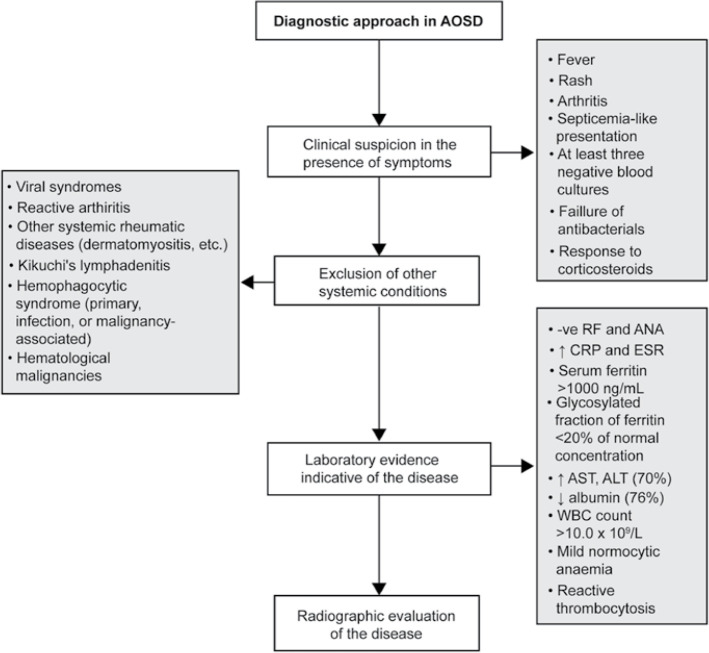
Diagnostic algorithm for AOSD. −ve: negative; ALT: alanine aminotransferase; ANA: antinuclear antibodies; AOSD: adult-onset Still’s disease; AST: aspartate aminotransferase; CRP: C-reactive protein; ESR: erythrocyte sedimentation rate; RF: rheumatoid factor; WBC: white blood cell. [Source: Kontzias A, Efthimiou P, Drugs, 2008;68(3):319–337]

AOSD accounts for 15–20% of all FUO cases.^76–78^ To differentiate patients with fever due to AOSD from those with FUO due to other causes, a simple algorithm with a sensitivity of 93.7% and a specificity of 95.4% was developed^79^; this algorithm includes two clinical (arthralgia and sore throat) and two laboratory (neutrophilia and serum ferritin level ≥5 × ULN) parameters, the presence of which strongly supports the diagnosis of AOSD in patients with FUO. Kontzias and Efthimiou^49^ have proposed a diagnostic algorithm (**Figure 2**) that emphasises that atypical presentations may occur in AOSD and that symptoms such as a high spiking fever, evanescent rash, and arthritis need not be present concomitantly at the onset of the disease. Recently, an algorithm with a points-based score (7 points), with a sensitivity of 92.5% and a specificity of 93.3%, was proposed by Daghor-Abbaci et al.^80^ Patients eligible for this algorithm should have at least two of the following criteria: fever ≥ 39°C, arthralgia or arthritis, pharyngitis, neutrophils percentage ≥80% or NLR ≥ 4, or ferritin > N.

## TREATMENT OPTIONS

### Current Treatment Strategy for Still’s Disease

The goal of treatment for Still’s disease is to achieve sustained remission^81^ by successfully controlling inflammation and, thereby, alleviating symptoms, reducing the risk of new flares, and preventing irreversible joint damage and life-threatening complications.

#### Conventional Treatments

Conventional treatments for Still’s disease involve the use of non-steroidal anti-inflammatory drugs (NSAIDs), corticosteroids, and conventional disease-modifying anti-rheumatic drugs (cDMARDs) such as methotrexate.^78,82– 84^ However, data reflects the efficacy achievable with these agents was not satisfactory in at least 30–40% of patients, particularly in controlling systemic and more severe manifestations, highlighting the need for other treatment options including targeted therapies.^82–85^

#### Biologics

Biologic agents that inhibit proinflammatory cytokines such as IL-1, IL-6, tumour necrosis factor (TNF)-α and IL-18 have been shown to interfere with the exacerbated inflammatory response in both sJIA and AOSD.^43,73^

### TNF-α Inhibitors

Anti-TNF agents such as etanercept, adalimumab and infliximab have shown limited efficacy in sJIA when compared with other JIA categories^43^ and low-to-moderate efficacy in refractory AOSD, particularly in the chronic articular form of the disease.^86–91^ Further, data from a case series showed that two patients, one on etanercept^92^ and the other on adalimumab,^93^ developed MAS. In AOSD patients with a predominant articular pattern, TNF inhibitors have been considered as a treatment option.^94^

### IL-6 Inhibitors

Tocilizumab (an IL-6 receptor inhibitor) was approved for the treatment of sJIA by both the European Medicines Agency (EMA) and the US Food and Drug Administration (FDA) in 2011. Tocilizumab has demonstrated efficacy in treating both systemic and articular features.^95,96^ Data indicate that treatment with tocilizumab in patients with AOSD led to improvements in systemic symptoms, ranging in duration from 2 months to 1 year.^97,98^ Limited studies exist for the use of tocilizumab in patients with AOSD, and a meta-analysis published in 2019 included only 10 retrospective studies of 147 patients.^98^ Data from a registry-based study^99^ that evaluated the efficacy and safety of tocilizumab in a cohort of patients with AOSD refractory to several therapies, including cDMARDs and other biologic agents showed that tocilizumab significantly decreased the Pouchot score and controlled disease activity. The median Pouchot score significantly decreased throughout the study period (*p*=0.001) with a significant difference between baseline and follow-up assessment at 6 months (*p*=0.003) and between baseline and the last follow-up assessment (*p*=0.032). Similarly, CRP, erythrocyte sedimentation rate (ESR) and serum ferritin levels significantly decreased between baseline and the last follow-up assessment.^99^ In addition to demonstrating efficacy, reports have shown a corticosteroid-sparing effect and acceptable safety profile with tocilizumab. However, liver dysfunction, MAS, hypertension, and dyslipidaemia have been reported as adverse events (AEs) with tocilizumab.^97^

In a case report of a single patient with AOSD,[100] treatment with the IL-6 receptor antagonist sarilumab at a dose of 200 mg every 2 weeks for 3 months led to normalisation of CRP levels, neutrophil counts, and ferritin levels, along with tapering of the methylprednisolone dosage.

### IL-1 Inhibitors

Anakinra, an IL-1 inhibitor, was approved for the treatment of sJIA in Australia in 2015,^101^ Still’s disease by the EMA in 2018^102^ and AOSD in the UK in 2021.^103^ Reports have shown that treatment with anakinra resulted in the resolution of symptoms and normalisation of inflammatory markers within 2–4 weeks in patients with refractory AOSD.^104–107^ Furthermore, in patients with AOSD receiving anakinra, tapering or discontinuation of corticosteroids has also been reported.^108^ Recently, Schanberg et al.^109^ reported the efficacy and safety data of anakinra in patients with Still’s disease across all age groups (<16 or ≥16 years at disease onset). All six patients randomised to anakinra achieved an American College of Rheumatology (ACR) 30 response with the absence of fever at Week 2 versus none in the placebo group (*p*=0.0022). In addition, patients who received anakinra achieved early onset of efficacy along with a decrease in CRP and ferritin levels (Week 1), with no unexpected safety findings.

Vastert et al.^110^ reviewed the effects of the IL-1 pathway blockade by anakinra in 27 studies in patients with either sJIA or AOSD. The authors observed that 23–88% of patients with sJIA achieved either an ACR paediatric response of ≥50% improvement (ACR Pedi 50) or clinically inactive disease. In patients with AOSD, response rates ranged from 50%–100% after a follow-up of 3–12 months. The variable efficacy response rates noted across studies could have been due to heterogeneity in disease duration and in treatment history in the different patient populations. Corticosteroid tapering and/or discontinuation was also reported across the analysed studies.^110^ The most common and consistently reported treatment-related adverse drug reaction was injection-site reactions. Injection-site reactions usually appeared within 2 weeks of initiation of anakinra treatment but tended to disappear within 4–6 weeks during continued treatment.^102^

The timing of anakinra therapy in sJIA could be one of the reasons for a varied response.^111^ Results of a retrospective multicentre study^112^ suggested that the use of anakinra as a first-line treatment early in the course of sJIA led to rapid resolution of systemic symptoms and prevented refractory arthritis in almost 90% of patients. Anakinra was well tolerated, although injection-site reactions were common. Four patients who received anakinra had MAS, but a causal association could not be established, and all patients continued anakinra therapy. Ter Haar et al.^113^ have adopted the treat-to-target approach in sJIA using anakinra as first-line monotherapy. Anakinra was tapered after 3 months and subsequently stopped in patients who achieved inactive disease. After 1 year of therapy, 76% of patients had inactive disease and 52% had inactive disease without medication, confirming the efficacy of first-line treatment with anakinra. The 5-year follow-up data showed that 96% of patients had inactive disease; of these, 75% achieved inactive disease while not receiving medication. The promising results of anakinra monotherapy as first-line treatment for sJIA guided researchers to initiate biologic therapy early in the disease course.^112^

In 2020, Vitale et al.^114^ reported no significant difference in clinical and therapeutic outcomes among patients with AOSD who had received early treatment with anakinra (ie, within 6 months of disease onset) versus delayed treatment (ie, within 6–12 months of disease onset). However, the authors observed a significant improvement in systemic score at 6-month assessment and swollen joints at 3-month assessment with early versus delayed treatment. Similarly, the corticosteroid-sparing effect, assessed at visits at 6- and 12 months, in patients who received early anakinra treatment was significantly better than in with those who received delayed treatment.

Rilonacept, an IL-1 inhibitor administered as a weekly subcutaneous injection, blocks the signalling of IL-1β by preventing its interaction with cell surface receptors.^115^ The efficacy and safety of rilonacept was assessed in 24 patients with sJIA during a 23-month, open-label treatment after a 4-week double-blind placebo-controlled phase.^116^ No significant difference in the adapted ACR Pedi 30 response was observed between the rilonacept and placebo arms at Week 4. However, at 3 months, 78.3%, 60.9%, and 34.8% of patients achieved adapted ACR Pedi 30/50/70 responses, which were maintained throughout the study. No serious drug-related AEs were reported, except for injection-site reactions, which was the most commonly reported AE. Petryna et al.^117^ described three cases of refractory AOSD, wherein rilonacept treatment led to an improvement in arthritic symptoms and in the tapering of prednisone. No AEs were reported in these cases.

Canakinumab was approved by the FDA and EMA in 2013 for the treatment of sJIA.^118,119^ Based on the concept that sJIA and AOSD are part of the same disease spectrum – including both the juvenile and adult-onset forms – and based on data from the CONSIDER trial,^120^ canakinumab was approved by the EMA and FDA for the treatment of active AOSD in 2016 and 2020, respectively.^118,119^ Two pivotal phase 3 trials demonstrated the efficacy and safety of canakinumab in sJIA patients with active systemic features.^121^ In both trials, infections were most frequent in the canakinumab group than in the placebo group. MAS was reported in seven patients, including two patients in the placebo group. Ruperto et al.^122^ and Brunner et al.123 reported a rapid and sustained response to canakinumab treatment in sJIA patients. Ruperto et al.^122^ reported that 25% (n=44) of patients with sJIA received at least three consecutive reduced doses of canakinumab of 2 mg/kg. Of these, 59% (n=26) of patients remained on a sustained reduced canakinumab dose until the end of the study with a median follow-up of 25 months.

To analyse treatment responses to canakinumab in different age groups, data were pooled from four studies on sJIA, where patients were grouped: children (aged 2 to <12 years), young adolescents (12 to <16 years) and older adolescents/young adults (≥16 years).^124^ Canakinumab was administered at 4 mg/kg every 4 weeks. The improvements observed in the systemic and arthritic components of the disease in older adolescents and young adults support the concept of a Still’s disease continuum. The clinical efficacy and safety profiles of canakinumab were comparable across the three age groups. The CONSIDER trial was terminated prematurely due to recruitment issues arising after marketing authorization of canakinumab.^118^ At Week 12, a higher proportion of patients in the canakinumab (66.7%) versus placebo (41.2%) group achieved an improvement in DAS-28 with an ESR of >1.2. The primary endpoint was not met. But treatment of patients with AOSD using canakinumab led to a statistically significant improvement versus placebo in several outcome measures, including ACR30/50/70 response rates. The safety profile of canakinumab in AOSD patients enrolled in the CONSIDER study^120^ was similar to that observed in sJIA patients.

In a retrospective, longitudinal, multicentre study from Greece comprising 50 adult patients with refractory Still’s disease,^125^ canakinumab showed long-lasting efficacy, with 78% of patients achieving a complete response within a median of 3 months irrespective of their age at disease onset. In addition, the use of corticosteroids was tapered in 51% (21/41) of patients treated. Canakinumab was well tolerated by most patients in the study; 10 (20%) developed infections and 3 (6%) had leukopenia related to canakinumab. To date, this has been the largest real-life cohort of adult patients with refractory Still’s disease treated with canakinumab. Additionally, a real-life evidence study from Italy^126^ that enrolled nine patients with AOSD found that treatment with canakinumab led to the resolution of clinical manifestations in the majority (8/9; 88.9%) of patients, a significant improvement of arthritic symptoms and systemic severity score and a steroid-sparing effect. None of the patients experienced any AEs during the follow-up period.

Moreover, data from case series have demonstrated the successful use of canakinumab in the treatment of patients with AOSD refractory to cDMARDs or other IL-1 inhibitors.^94,127–132^ Banse et al.^128^ described the occurrence of MAS after two injections of canakinumab in a patient with AOSD; this suggests a potential causal relationship even though MAS is a well-known complication of AOSD. In a cross-sectional observational study involving the off-label use of anti–IL-1 treatments in France, canakinumab was used as second-line treatment after anakinra therapy in 21 of 25 patients. Of the 25 patients, 2 with AOSD received canakinumab 150 mg every 4 or 8 weeks; the patient treated with canakinumab every 8 weeks and on concomitant corticosteroid therapy achieved complete remission, while the other did not improve.^105^ Safety data from the study indicates that mild respiratory infection (17%), liver toxicity (9%) and injection-site reactions (4%) were the AEs reported in patients who had received canakinumab for other off-label indications. Severe infection was the most commonly reported serious AE.

Brachat et al.^30^ analysed the gene-expression profiles of patients with sJIA who had responded to canakinumab treatment and observed the downregulation of many innate immunity-related genes, such as those related to inflammation and their corresponding proinflammatory markers.

Klein et al.^133^ analysed data on safety from the German Biologics JIA Registry of 260 patients with sJIA who had been treated with etanercept, tocilizumab, anakinra, and/or canakinumab. The incidence of MAS was highest in patients treated with canakinumab (3.2/100 patient-years [PY]), followed by tocilizumab (2.5/100 PY), anakinra (0.83/100 PY), and etanercept (0.5/100 PY). Overall, an acceptable safety profile was observed in all treatment cohorts. Cabrera et al.^134^ conducted a systematic review and meta-analysis of efficacy and safety data from 19 randomised controlled trials involving 1458 patients; the authors concluded that biological agents were efficacious, particularly for patients with systemic-onset JIA versus non-systemic JIA, and they were not associated with serious AEs.

In general, treatment with IL-1 inhibitors led to the resolution of clinical signs and symptoms along with a favourable safety profile in both sJIA and AOSD supporting the concept that both entities, while distinct, are part of the same disease spectrum.

### Other Potential Therapeutic Agents

Treatment with tadekinig alfa, a recombinant human IL-18−binding protein, showed 50% response rates in CRP reduction, normalisation of ferritin levels and a ≥20% reduction in the count of swollen and tender joints, along with a favourable safety profile in patients with AOSD.^135^ In a case study by Ladhari et al.^136^ administration of baricitinib, a Janus kinase (JAK) 1 and 2 inhibitor, in combination with anakinra to a patient with refractory AOSD led to the resolution of clinical symptoms; remission was observed within 9 months. Baricitinib administration to two more patients with refractory AOSD provided mixed results^137^: one patient remained in remission for 15 months, while the other did not show any improvements. In another case series, a patient with AOSD and pre-existing myelodysplasia treated with baricitinib remained in remission for 9 months but developed *Pneumocystis jirovecii* pneumonia at 7 months.^137^ Hu et al.138 reported the efficacy of tofacitinib, a JAK1/3 inhibitor, in patients with refractory AOSD. Out of 14 patients studied, 7 achieved complete remission with a corticosteroid-sparing effect and 6 achieved partial remission. In another study, tofacitinib (5 mg/day) was administered to a patient with AOSD complicated with MAS who had previously received tocilizumab and then cyclophosphamide. Improvements were observed in serological parameters and there was no flare of AOSD even after glucocorticoid tapering.^139^ A retrospective study140 of JAK inhibitors in patients with difficult-to-treat AOSD and sJIA (N=9; baricitinib, n=5 [one switched to upadacitinib later]; ruxolitinib, n=2; tofacitinib, n=2) showed a mixed response, with two complete remissions, three partial remissions, and four treatment failures, reflecting the varied clinical presentation and course of the disease. In an ongoing, open-label, single-arm study,^141^ the efficacy and safety of oral 5-aminolevulinic acid plus sodium ferrous citrate are being analysed in glucocorticoid-dependent patients with AOSD.

In sJIA patients without MAS, American College of Rheumatology 2021 guidelines^142^ conditionally recommend use of NSAIDs or biologic DMARDs (IL-1 and IL-6 inhibitors) as first-line monotherapy. For patients with sJIA with MAS, glucocorticoids are conditionally recommended as part of initial treatment, IL-1 or IL-6 inhibitors monotherapy or combination of csDMARDs for patients with inadequate response to or intolerance of NSAIDs and/or glucocorticoids.

Corticosteroids are used as the first-line treatment for AOSD. In patients who failed to achieve remission, or who are dependent on steroids for symptomatic control, a DMARD (methotrexate or azathioprine) is added to the therapy after diagnosis of AOSD is confirmed.^143^. Patients with polycyclic systemic pattern or refractory AOSD who failed to achieve remission may require further therapy with biologics. Currently canakinumab and anakinra are approved for the treatment of AOSD.^143^

## SUMMARY

sJIA and AOSD are rare systemic autoinflammatory disorders. Comparisons of genetics, pathogenesis, clinical presentation, and the natural course of each condition support the concept of a Still’s disease continuum. A pivotal role is played by the innate immune system in the pathogenesis of both conditions, with the involvement of major proinflammatory cytokines, including IL-1β, IL-18, and IL-6. Recent advances in the management of sJIA have introduced the concept of a “window of opportunity” with inactive disease or minimal disease activity as treatment targets, aiming for comprehensive disease control.^144^ Still’s disease management is evolving, with greater use of targeted therapies – including in the first-line setting, based on their ability to achieve and maintain clinical remission – to allow for the tapering or withdrawal of corticosteroids to potentially avoid complications.

## Data Availability

Not applicable.

## Abbreviations:

AA: amyloid A;
ACR: American College of Rheumatology;
AOSD: adult-onset Still’s disease;
cDMARDs: convention drugs;
CRP: C-reactive protein;
EMA: European Medicines Agency;
ESR: erythrocyte sedimentation rate;
FDA: Food and Drug Administration;
FUO: fever of unknown origin;
GF: glycosylated ferritin:
IL: interleukin;
IL-1RAP: Interleukin-1 receptor accessory protein;
ILAR: International League of Associations for Rheumatology;
MAS: macrophage activation syndrome;
NETs: neutrophil extracellular traps;
NLR: neutrophil-to-lymphocyte ratio;
sJIA: systemic juvenile idiopathic arthritis;
sTREM-1: soluble form of triggering receptor expressed on myeloid cells-1;
ULN: upper limit of normal
